# Incidental carcinoma of the prostate gland presenting with initial manifestation of disseminated intravascular coagulopathy (dic) in a middle aged man: a case report

**DOI:** 10.1186/1757-1626-2-144

**Published:** 2009-09-29

**Authors:** Ayo Abdulkadir Salako, Olukayode Adeolu Arowolo, Emmanuel Abidemi Omonisi, Adewale Oluseye Adisa, Nicholas Akinwale Titiloye, Kayode Adelusola

**Affiliations:** 1 Department of Surgery, Obafemi Awolowo University, Ile - Ife, Osun State, Nigeria; 2 Department of Morbid anatomy & Forensic medicine, Obafemi Awolowo University, Teaching Hospital, Ile - Ife, Osun State, Nigeria

## Abstract

**Background:**

Incidental carcinoma of the prostate gland is a common clinical problem among elderly males but this malignancy presenting initially with features of Disseminated Intravascular Coagulopathy (DIC) in the African blacks is rare. Disseminateded intravascular coagulathy is the most frequent coagulation disorder in patients with prostate cancer, However DIC as a first manifestation of prostate cancer is unusual.

**Case report:**

This paper reports a case of a 56 year old Nigerian civil servant who presented initially with clinical features of DIC characterised by bleeding from multilple orifices but was subsequently diagnosed at autopsy to be infiltrating adenocarcinoma of the prostate.

**Conclusion:**

This rare case of DIC should be considered especially in elderly men when no other cause can be found for coagulopathy.

## Introduction

Carcinoma of the prostate is the most common cancer in men and constitutes the third most frequent cause of death from cancer in the males, following only cancer of the lung and colorectal cancer [1-3]. With the discovery of PSA (Prostatic Specific Antigen) tumor marker, prostate cancer is now diagnosed early in many asymptomatic patients on routine medical examination or screening in the developed world [2,3]. However in many underveloped countries, patients present late usually with symptoms of local invasion characterised by gross heamaturia, obstructive urinary symptoms and low back pain[2]. Prostate carcinoma may be present as a localized lesion or as an invasive lesion with systemic metastasis. Distant spread may occur by the lymphatic system or hematogeneous route, with osseous metastasis constituting the most common form of hematogeneous spread[3].

Disseminated Intravascular Coagulation (DIC) as an initial presentation of prostatic Adenocarcinoma is rare[3-5]. We report a case of 56-year old man with metastatic prostatic carcinoma incidentally diagnosed at autopsy presenting with initial clinical manifestation of disseminated intravascular Coagulopathy.

## Case Presentation

A 56-year -old black African man. He is a civil servant of yoruba ethnicity extraction in Nigeria and weighs 65 kilogramm with height of 157 cm. He presented to the accident & emergency unit of Obafemi Awolowo University Teaching Hospital, Ile-Ife, Nigeria with a day history of epitaxis and massive upper gastrointestinal bleeding. He was estimated to have lost about two litres of blood at presentation and he was also in heamodynamic instabilty. There was associated passage of meleana stool a day before presentation. He was not to have peptic ulcer disease in the past and there was no history of abuse of steroid or non steroidal anti inflammatory drugs (NSAID).

The patient in addition had significant obstructive urunary syptom of straining, poor urinary stream and overlow incontinence. The patient does not smoke nor drink alcohol.

On examination, we found middle aged man, in acute distress, diaphoretic and restless, he was profoundly pale but anicteric and acyanosed. There were no palpable peripheral lymph node and no pedal oedema.

On Examination of the cardiovascular system the pulse was 120/min small volume and regualr with blood pressure of 100/70 mmhg. Examination of the abdomen showed epigastric tenderness with hepatomegaly and bladder fullness. Rectal examination revealed an enlarged nodular prostate.

Investigations carried out include pack cell volume of 13%, blood chemistry were essentially normal.

A clinical diagnosis of sever upper gastrointestinal bleeding probably due chronic duodenal ulcer was made, epistaxis was tought to be due to massive upper gastrointestinal bleeding.

The patient was resucitated, He had three pints of fresh frozen plasma and another three pints of fresh whole blood and intravenous fluids. Intravenous Ranitidine 50 mg twelve hourly, he had urethral catheter passed to monitor urine output. He was planned for upper gastro-intestinal endoscopy after adequate resucitation but his condition deteriorated and died on the day of admission. Also no radiological investigation was done as the patient was not fully resucitated to allow any radiologicalm investigation before his demise.

## The Autopsy Findings

At autopsy, the body was severally pale; fragments of clotted blood were seen in his nostrils.

The serous cavities contained bloody fluids; 80 mls in the pericardial cavity, 2 Litres in the peritoneal cavity, 600 mls in the right pleural cavity and 400 mls in the left pleural cavity.

**Figure 1:**
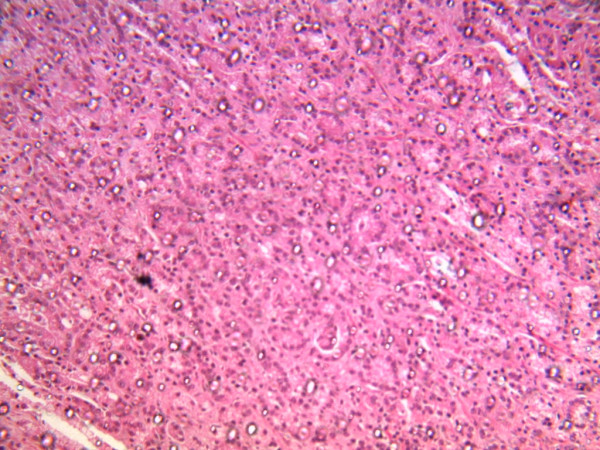
**Microscopic appearance of prostatic carcinoma:** The field shows micro - nacini of small malignant cells infiltrating the prostatic stroma in areas (H & E × 100).

The review of the organ systems revealed an enlarged and firm prostate weighed 150 g with multiple nodules of various sizes and shapes seen mostly at the median lobe. Cut surfaces of the prostate shows nodular surfaces with golden yellow nodules. Microscopic sections of the prostate showed an infiltrating Adenocarcinoma Gleason grade V. (Figure 1)

The urinary bladder was distended and filled with 1.2 L of concentrated urine. Cut surface of the urinary bladder showed increased trabeculation with hyperemia of the bladder mucosa and narrowing of the neck of the bladder. Microscopic sections of the urinary bladder showed chronic non-specific cystitis

Kidneys were enlarged, the right weighed 210 g and the left kidney weighed 200 g. Microscopic sections of both kidneys show features consistent with acute tubular necrosis.

**Figure 2:**
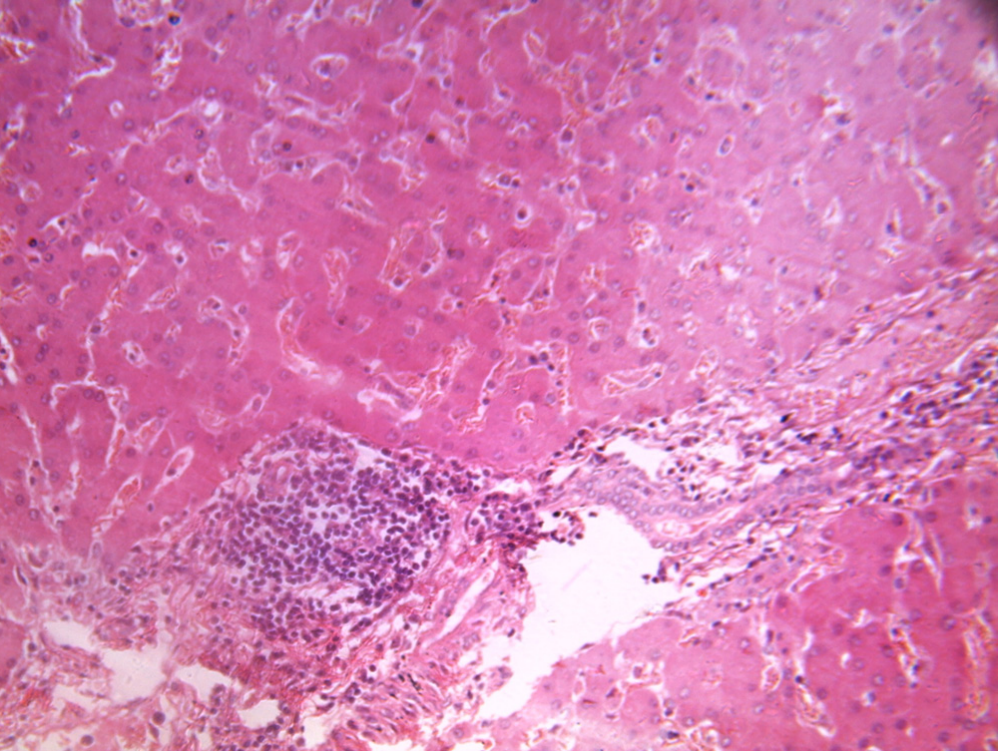
**liver metastasis at the portal hepatis area from advanced disseminated prostatic cancer (H & E × 16)**.

The liver weighed 1500 g, multiple greyish white nodules of various sizes and shapes with umbilcation on their surfaces were seen. Cut surfaces of the liver were flattened and the edges were sharp. The nodules do not extend into the liver parenchyma. Similar nodules were seen in the rectum. The remaining parts of the gastrointestinal system were unremarkable. Microscopic sections of the liver showed evidence of metastatic adenocarcinoma (Figure 2) showed metastatic deposit from the prostate cancer in the area of portal hepatis.

**Figure 3:**
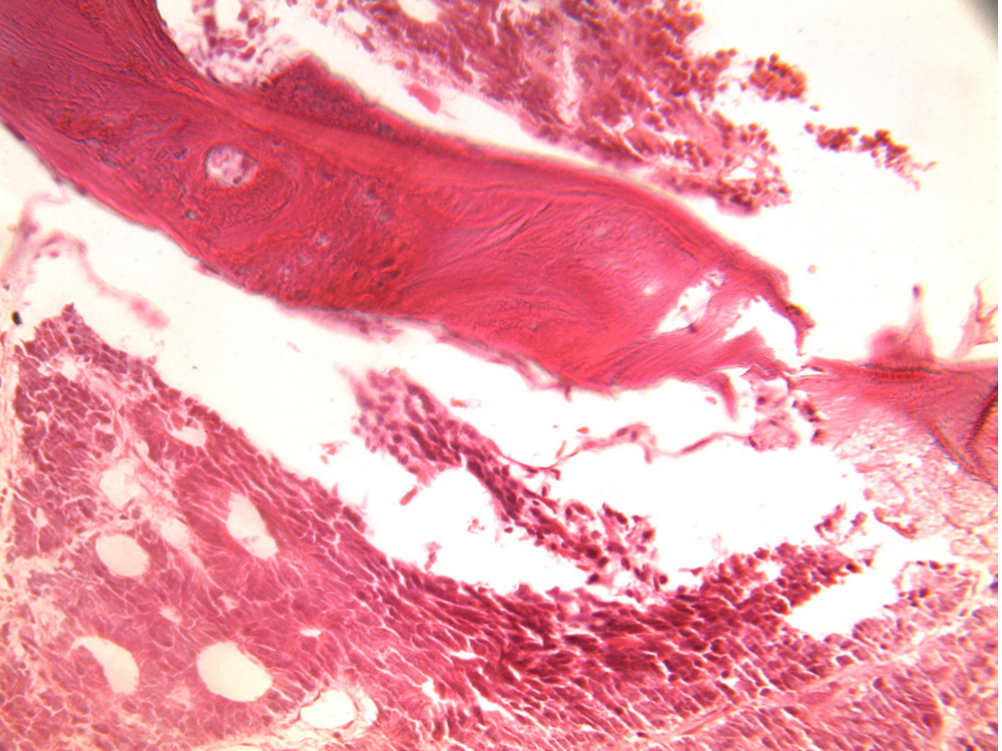
**Metastatic adenocarcinoma to the bone marrow from the disseminated prostatic tumour (H & E × 25)**.

The tracheobronchial tree, heart and musculoskeletal system were essentially unremarkable. *T*here were also evidence of metastasis to the bone as shown in (Figure 3)

## Discussion

Prostate cancer is an important growing health problem, presenting a challenge to the Urologists, Pathologists, Radiologists and Oncologists [6-9]. Prostate cancer is the most common non- dermatologic cancer, yet despite this frequent occurrence, the clinical course is often unpredictable [10,11]. Most prostate cancers are slow growing however some are aggressive, with a rapidly worsening course. Many men are found to have incidental microscopic foci of prostatic cancer at postmortem examination[2].

Disseminated Intravascular Coagulopathy (DIC) is an acquired coagulation disorder that may occur in a wide variety of clinical conditions. Confirmation of the diagnosis of DIC should always prompt a search for underlying medical disorders, including sepsis, severe trauma. Solid and hematological malignancies, obstrectric complication and vascular disorders. DIC revealing a prostatic adenocarcinoma is rare. Most of the cases are limited to biological abnormalities [12-14]. Hypercoagulable states associated with malignancy resulting in thrombocytopenia and DIC are well recognized. [3] Tissue thromboplastins derived from the tumor cells that are exposed to the circulation are believed to be important in the pathophysiology[6]. The manifestation of DIC associated with prostatic cancer can range from being a subclinical marker of disease [3,4] to overt bleeding after minor to moderate trauma [5]. We reported this case to increase the awareness that prostatic adenocarcinoma may present initially with features of DIC. Therefore, high index of suspicions is the watch word.

## Conclusion

This report highlighted the very rare presentation of prostatic carcinoma with upper gastrointestinal bleeding secondary to Disseminated intravascular coagulation (DIC). Abscence of gastric and Duodenal ulcer at autopsy and confirmation of prostatic carcinoma at histology confirms the malignant prostate as the origin of the widespread DIC. The fact that there were histological evidence of metastasis in the bone and liver confirms the advance nature of the prostate cancer as distinct from cases of incidental findings at postmortem examination. This rare case of DIC should be considered especially in elderly men when no other cause can be found for coagulopathy.

## Consent

Written informed consent was obtained from the living next of kin of the patient for publication of this case report and accompanying images. We could not obtained direct consent from the patient because the patient died before this report was written. A copy of the written consent from the next of kin is available for review by the Editor-in-Chief of this journal.

## Competing interests

The authors declare that they have no competing interests.

## Authors' contributions

AAs, AAA and AOA are involved in the primary management of the patient and also in writting of the manuscript. AEO, NAT and KA are the patologist who performed autopsy and histological examination of the prostatic slide. All authors read and approved the final manuscript.
